# Machine learning-based identification of the risk factors for postoperative nausea and vomiting in adults

**DOI:** 10.1371/journal.pone.0308755

**Published:** 2024-08-15

**Authors:** Hiroshi Hoshijima, Tomo Miyazaki, Yuto Mitsui, Shinichiro Omachi, Masanori Yamauchi, Kentaro Mizuta

**Affiliations:** 1 Division of Dento-Oral Anesthesiology, Tohoku University Graduate School of Dentistry, Miyagi, Japan; 2 Graduate School of Engineering, Tohoku University, Miyagi, Japan; 3 Department of Anesthesiology, Tohoku University Hospital, Miyagi, Japan; Taipei Medical University, TAIWAN

## Abstract

Postoperative nausea and vomiting (PONV) is a common adverse effect of anesthesia. Identifying risk factors for PONV is crucial because it is associated with a longer stay in the post-anesthesia care unit, readmissions, and perioperative costs. This retrospective study used artificial intelligence to analyze data of 37,548 adult patients (aged ≥20 years) who underwent surgery under general anesthesia at Tohoku University Hospital from January 1, 2010 to December 31, 2019. To evaluate PONV, patients who experienced nausea and/or vomiting or used antiemetics within 24 hours after surgery were extracted from postoperative medical and nursing records. We create a model that predicts probability of PONV using the gradient tree boosting model, which is a widely used machine learning algorithm in many applications due to its efficiency and accuracy. The model implementation used the LightGBM framework. Data were available for 33,676 patients. Total blood loss was identified as the strongest contributor to PONV, followed by sex, total infusion volume, and patient’s age. Other identified risk factors were duration of surgery (60–400 min), no blood transfusion, use of desflurane for maintenance of anesthesia, laparoscopic surgery, lateral positioning during surgery, propofol not used for maintenance of anesthesia, and epidural anesthesia at the lumbar level. The duration of anesthesia and the use of either sevoflurane or fentanyl were not identified as risk factors for PONV. We used artificial intelligence to evaluate the extent to which risk factors for PONV contribute to the development of PONV. Intraoperative total blood loss was identified as the potential risk factor most strongly associated with PONV, although it may correlate with duration of surgery, and insufficient circulating blood volume. The use of sevoflurane and fentanyl and the anesthesia time were not identified as risk factors for PONV in this study.

## Introduction

Postoperative nausea and vomiting (PONV) is a common adverse effect after general anesthesia, and with reported to affect 20–30% of post-surgical patients [1,2]. PONV is also one of the most common causes of patient dissatisfaction after general anaesthesia [3] and increases medical costs because of the requirement for prophylaxis and treatment, length of hospital stays, and readmissions [4]. Therefore, it is crucial to identify and prevent risk factors for PONV [5]. Some patient-related, surgery-related, and anesthesia-related risk factors have been associated with higher incidences of PONV. The patient-related risk factors include female sex, younger age, non-smoker, history of motion sickness, and a previous episode of PONV [6–9]. Surgery-related risk factors include intra-abdominal surgery, ear surgery, testicular fixation, and tonsillectomy [10]. Anesthesia-related risk factors include the use of inhalation anetshtetics [6,11], postoperative opioid use, and the duration of general anaesthesia [10,12]. Although perioperative use of antiemetics (5-HT_3_ receptor antagonists, dopamine D_2_ receptor antagonists, neurokinin receptor 1 antagonist, and corticosteroids) or the use of propofol as the maintenance of general anesthesia have been suggested to be effective in preventing PONV, its prevention remains difficult [6,13] because various patient-related, surgery-related, and anesthesia-related risk factors interact each other in a complex manner to increase the frequency of PONV [14].

Recently, artificial intelligence (AI) has made remarkable technological progress [15,16], and its application in the healthcare field is also advancing [17,18]. So-called deep learning techniques are increasingly used in machine learning-based AI. Unlike conventional machine learning, deep learning is characterized by AI learning the difference between samples and selecting the correct answer. In this way, AI can independently recognize changes that are outside human perception and establish AI-specific identification methods.

The aim of this study was to identify risk factors for PONV by applying machine learning-based AI.

## Material and methods

### Study setting and variables

The requirement for written informed consent was waived by the IRB. This study was approved by the Tohoku university’s Institutional Review Board (IRB #2020-3-032) and written informed consent was obtained from all subjects participating in the trial. The trial was registered prior to patient enrollment at UMIN (UMIN000042562, Principal investigator: Hiroshi Hoshijima, Date of registration: November 26, 2020). The subjects were adult patients aged ≥20 years who underwent surgery under general anesthesia at Tohoku University Hospital for 10 years from 2010 to 2019. The following exclusion criteria were applied: age ≤ 19 years, emergency surgery, cardiac surgery, surgery with differential lung ventilation, electroconvulsive therapy, cesarean section, surgery with regional (spinal, epidural, or nerve block) anesthesia only, severe intraoperative or postoperative complications (cardiac arrest, severe arrhythmia, myocardial infarction, severe asthma, pulmonary embolism), admission to the postoperative intensive care unit (without extubation) and patients receiving intraoperative droperidol for PONV prophylaxis. Data were extracted from electronic medical records, anesthesia charts, and nursing records on surgical procedures. Extracted data include followings; (1) the episode of nausea, vomiting, and use of antiemetics within 24 hours after surgery. (2) Patient-related factors: sex, age, body mass index (BMI). (3) Time-related factors: duration of anesthesia (min), duration of surgery (min), and anesthesia start time [morning (8:30–12:00), noon (12:01–17:00, or evening (17:01–24:00)]. (4) Fluid-related factors; total infusion volume (ml), total blood loss and blood transfusion volume during surgery (ml). (5) Anesthetic agent-related factors: anesthesia method (inhalational or total intravenous anesthesia), amounts of inhalational anesthetics used for maintenance of anesthesia [desflurane (ml) and sevoflurane (ml)], number of propofol ampules (200 mg per ampule) used for induction of anesthesia, amount of propofol prefilled syringes (500 mg per syringe) used for induction and maintenance of anesthesia (mg; the amount was not the actual dose administered to the patients, but was calculated backwards from the number of prefilled syringes used), amount of fentanyl (0.1 or 0.25 mg per ampule) used (mg; the amount was not the actual dose administered to the patients, but was calculated backwards from the number of ampules used), number of remifentanil vials (2 or 5 mg per vial) used, amount of rocuronium (50 mg per vial) used (mg; the amount was not the actual dose administered to the patients, but was calculated backwards from the number of vials used), and amount of sugammadex (200 mg per vial) used (mg; the amount was not the actual dose administered to the patients, but was calculated backwards from the number of vials used). (6) Level of epidural anesthesia (cervical/thoracic, lumbar, or sacral). (7) Use of cardiovascular agents during anesthesia; numbers of dopamine prefilled syringes (20 mg/syringe), atropine ampules (0.5 mg per ampule), phenylephrine ampules (1 mg per ampule), and nicardipine ampules (2 or 5 mg per ampule) used during anesthesia. (8) Patient positioning during surgery: intraoperative patient positioning [lateral or prone]. (9) Laparoscopic or non-laparoscopic surgery.

### Machine learning modeling

We built a model that predicts the probability of PONV and performed an analysis with the model to reveal the impacts of items in the patient data. Then, we identified the risk factors of PONV according to the impacts. The model predicts the probability of PONV using the gradient tree boosting model [19] which is a widely used machine learning algorithm in many applications due to its accuracy and computational efficiency. The model consists of multiple decision trees and summarizes their prediction values, resulting in a final prediction probability. We implemented the model using the LightGBM framework [20]. We randomly split the patient data into training data (70%), validation data (20%), and test data (10%).

This study aims to identify risk factors of PONV using machine learning algorithms. Specifically, we used the Shapley values [21] to measure the impacts of the output of the LightGBM model. The Shapley value of an item represents the contribution of the item to the prediction value. Eq (1) defines the Shapley value *ϕ*_*i*_ for item *i* in model *f*. ℛ is the set of all items in the patient data, *P*^*R*^ is the set of features including item *i*. *M* is the number of items (*M* = 31 in this paper). Thus, the Shapley value for item *i* is the expected value over sets of items with and without item *i*. We use an approximation framework for the Shapley value calculation [22]. The theoretical background of the Shapley values is based on a model interpretation algorithm [23], which is the Shapley value used in the paper. We use the algorithm to measure the influence of each input factor on the prediction results. Thus, we identify the main factors that affect the PONV prediction. The Shapley value is from cooperative game theory. The output score is allocated to input members.


$$\phi_{i}=\sum_{R\in R}\frac{1}{M!}[f(P^{R})-f(P^{R}\setminus \{i\})]$$ (1)

## Results

### Eligible data

Data were obtained from 37,548 patients who underwent surgery under general anesthesia from a database at Tohoku University Hospital and met the inclusion criteria. Finally, data of 33,676 patients were obtained after excluding the data of 3,872 patients (defect data, use of droperidol, cesarean section, pulmonary differential lung ventilation, and more than two anesthetics were used to maintain general anesthesia) (S1 Fig). In this study, PONV occurred in 3,503 (10.4%) patients. The patient characteristics are shown in Table 1 and the types of surgery are shown in Table 2.

**Table 1:** Patients characteristics.

		Patients with of PONV (3503, 10.4%)	Patients without of PONV (30173, 89.6%)
Gender (M/F)		941 (26.9%)/2562 (73.1%)	15175 (50.1%)/14998 (49.9%)
Age (year)		54.0 ± 17.2	57.6 ± 17.2
Body Mass Index(BMI, kg/m^2^)		23.2 ± 4.64	23.3 ± 4.32
Duration of surgery (min)		183.5 ± 132.9	201.1 ± 173.0
Duration of anesthesia (min)		255.1 ± 147.9	274.9 ± 187.1
Total infusion volume (ml)		1521.6± 1094.8	1809.2± 1594.2
Total bleeding (ml)		183.7± 418.4	183.5± 1041.3
Total blood transfusion volume (ml)		51.2± 309.8	128.1± 927.8
Total urine volume (ml)		381.7± 483.9	287.8± 491.1
Laparoscopic surgery (n)		676	4,882
Non-laparoscopic surgery (n)		2,827	25,291
With epidural anesthesia (cervical or thoracic) (n)		806	7,572
With epidural anesthesia (lumbar) (n)		191	936
With epidural anesthesia (sacral) (n)		2	10
Anesthesic agents			
	Desflurane (ml)	78.7± 48.1	85.9± 63.1
	Sevoflurane (ml)	53.3± 35.4	49.3± 29.2
	Propofol (ampule) (n*)	1,194	10,211
	Propofol (prefilled syringe) (mg**)	1297.2± 748.4	1367.3± 881.4
	Fentanyl (mg**)	0.56± 0.53	0.53± 0.49
	Remifentanil (total number of vials used)	3,407	28,057
	Rocuronium (mg**)	72.9± 46.9	73.9± 54.1
	Sugammadex (mg**)	107.3± 107.1	109.9± 106.9
Cardiovaccular agents			
	Atropine (n*)	86	1,791
	Dopamine (n*)	915	5,830
	Nicardipine (n*)	260	2,689
	Phenylephrine (n*)	95	1,332

* Total unmber of ampules or prefilled syringe used in each group.

**The amount of drug used (mg) is not the actual dose administered to the patients, but is calculated backwards from the number of ampules/vials of the drug administered to the patients.

M; male, F; female.

**Table 2:** Type of surgery.

Type of surgery	Patients with of PONV (3,503)	Patients without of PONV (30,173)	Total (33,676)
Digestive*, vascular, and transplant surgery	1,245	10,015	11,260 (33.4%)
Oral surgery	386	3,335	3,721 (11.0%)
Gynecology	588	3,113	3,701 (10.9%)
Urology	249	3,130	3,379 (10.0%)
Orthopedics	439	2,862	3,301 (9.8%)
Otorhinolaryngology	221	2,850	3,071 (9.1%)
Neurosurgery	200	1,868	2,068 (6.1%)
Ophthalmology	33	1,161	1,194 (3.5%)
Plastic surgery	116	995	1,111 (3.3%)
Dermatology	26	844	870 (2.6%)

*Esophagus, stomach, liver, gallbladder, and pancreas surgery.

First, we evaluated the prediction model using three metrics: the true positive rate, the false positive rate, and the area under the curve. Thereafter, we compared this prediction model with a naïve (*k*-nearest neighbor [KNN]) model with *k* set to 3. The receiver-operating characteristic curves were shown in S2 Fig. According to the area under the curve, the prediction model outperformed the KNN model (0.77 vs 0.59). Therefore, our model successfully learned to predict PONV and is worth analyzing. Besides, We used the work [24] to calculate the 95% confidence intervals for the AUC. The results are shown in Table 3.

**Table 3:** Ablation study of the anesthesia duration.

	AUC	Lower	Upper
KNN (k = 3)	0.59	0.56	0.63
GBM	0.77	0.74	0.79

We then calculated the mean absolute SHAP (Shapley Additive exPlanations) values for each item by averaging absolute SHAP values over the test data (S3 Fig). A greater value represents a greater impact on the prediction of PONV.

### Results of contribution to PONV

**Fig 1:**
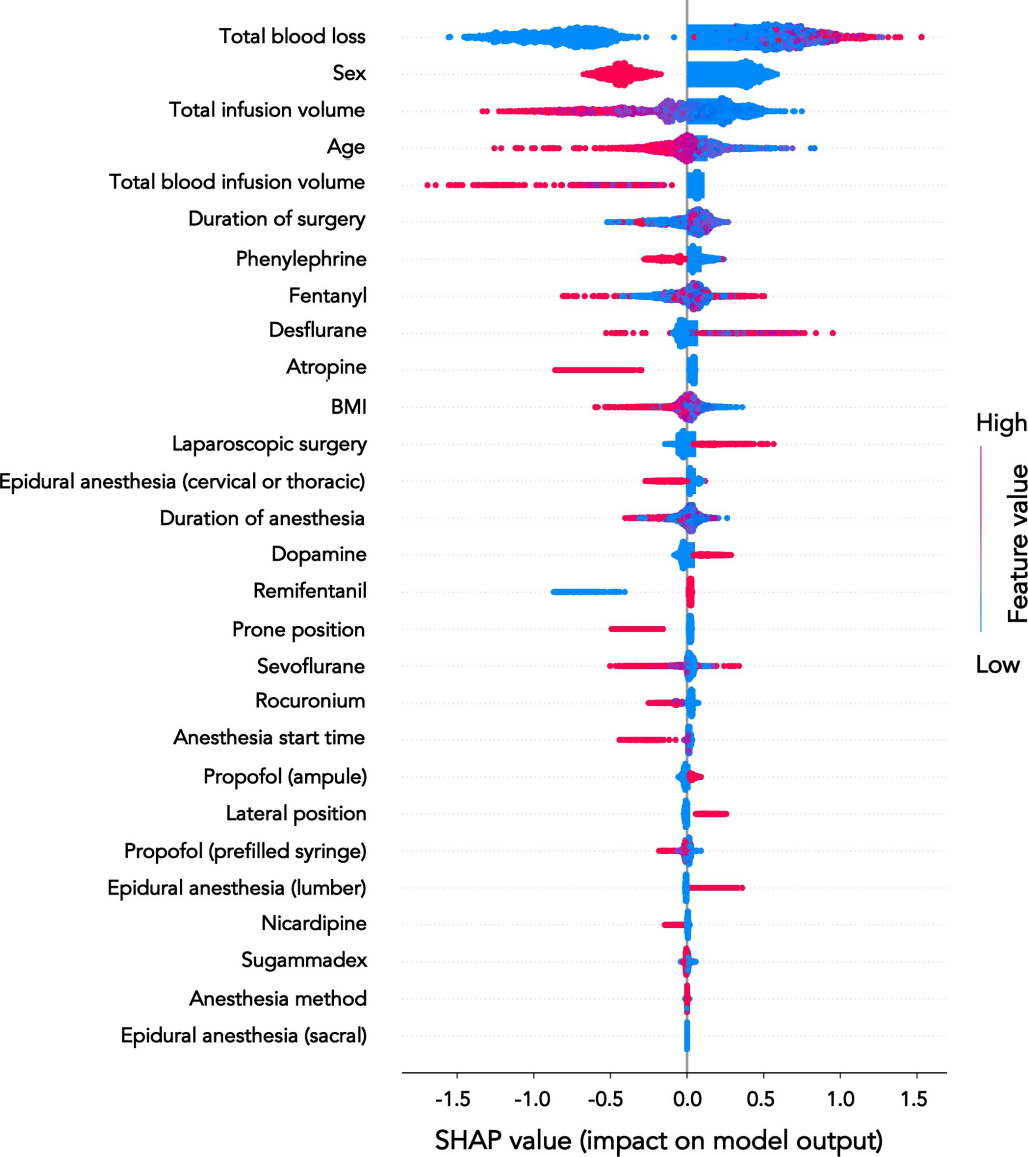
Summary of plot. The risk factors for PONV are plotted in descending order of relevance.

The contribution of each factor in the development of PONV is plotted in Fig 1. The item with the greatest ability to predict PONV was total blood loss during anesthesia, followed by sex, total infusion volume during anesthesia, and patient’s age. The factor with the lowest contribution to PONV was epidural anesthesia at sacral levels.

### Results of the risk factors for PONV

**Fig 2:**
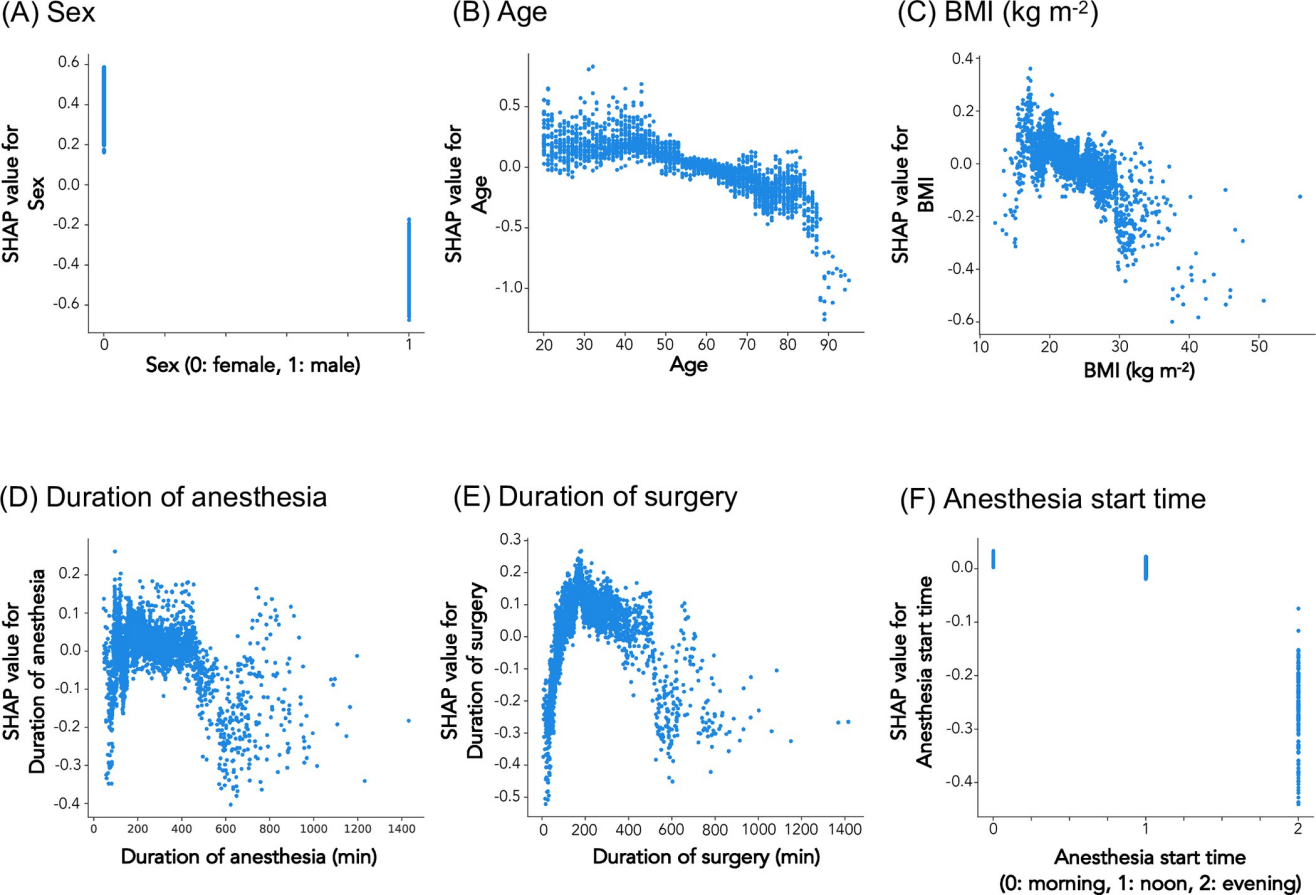
Relationship between patient-related and time-related risk factors and PONV in SHAP value. The X-axis represents (A) sex (0: female, 1: male), (B) age, (C) BMI (kg m^2–1^), (D) duration of anesthesia (min), (E) duration of surgery (min), and (F) anesthesia start time [morning (8:30–12:00), noon (12:01–17:00, or evening (17:01–24:00)]. The Y-axis represents the risk of PONV. SHAP value above 0 is related to the risk of PONV.

**Fig 3:**
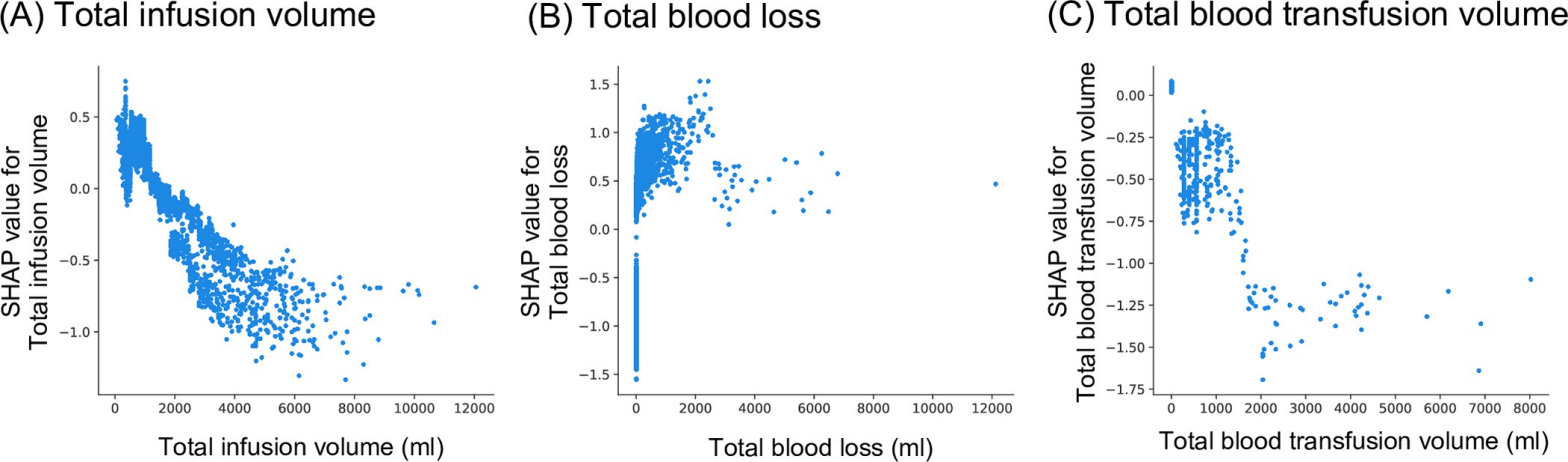
Relationship between fluid-related risk factors and PONV in SHAP value. The X-axis represents (A) total infusion volume (ml), (B) total blood loss (ml), and (C) total blood transfusion volume (ml). The Y-axis represents the risk of PONV. SHAP value above 0 is related to the risk of PONV.

**Fig 4:**
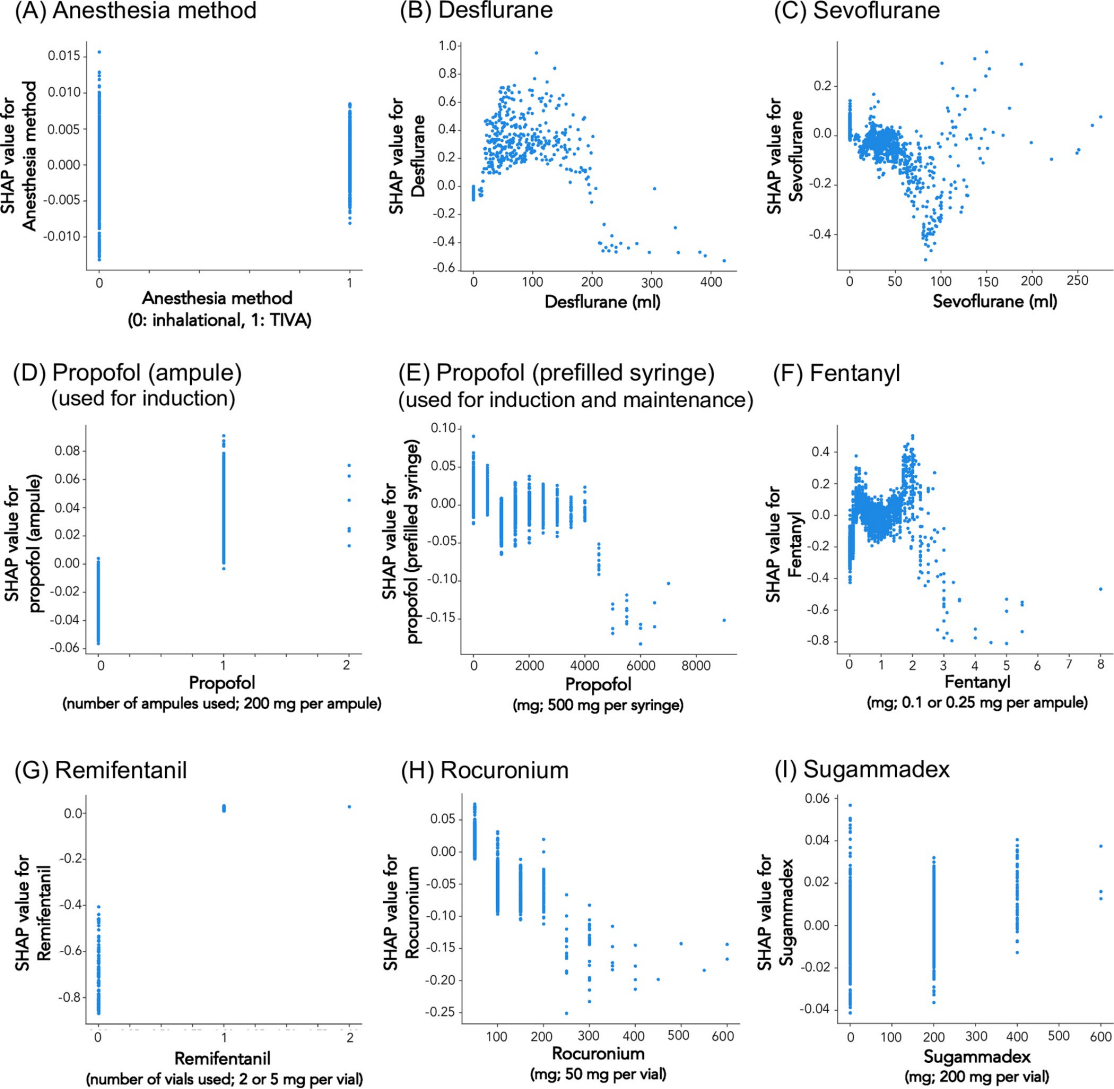
Relationship between anesthetic agent-related risk factors and PONV in SHAP value. The X-axis represents (A) anesthesia method (0: inhalational anesthesia, 1: total intravenous anesthesia), (B) use of desflurane for maintenance of anesthesia (ml), (C) use of sevoflurane for maintenance of anesthesia (ml), (D) number of propofol ampules (200 mg/ampule) used for induction of anesthesia, (E) amount of propofol prefilled syringes (500 mg/syringe) used for induction and maintenance of anesthesia (mg; the amount was not the actual dose administered to the patients, but was calculated backwards from the number of prefilled syringes used), (F) amount of fentanyl (0.1 or 0.25 mg/ampule) used (mg; the amount was not the actual dose administered to the patients, but was calculated backwards from the number of ampules used), (G) number of remifentanil vials (2 or 5 mg/vial) used, (H) amount of rocuronium (50 mg/vial) used (mg; the amount was not the actual dose administered to the patients, but was calculated backwards from the number of vials used), and (I) amount of sugammadex (200 mg/vial) used (mg; the amount was not the actual dose administered to the patients, but was calculated backwards from the number of vials used). The Y-axis represents the risk of PONV. SHAP value above 0 is related to the risk of PONV.

The analysis identified patient-related risk factors for PONV to be female sex (Fig 2A) and younger age (20–50 years; Fig 2B), and time-related risk factors to be the duration of surgery (60–400 min; Fig 2E). Fluid-related risk factors for PONV were total fluid infusion volume during anesthesia less than 1,000 ml (Fig 3A), total blood loss during surgery between 1–2,500 ml (Fig 3B), and no blood transfusion (Fig 3C). Anesthetic agent-related risk factors for PONV were the use of desflurane for maintenance of anesthesia (Fig 4B), and non-use of propofol prefilled syringe (Fig 4E). PONV was also associated with the use of dopamine (S4B Fig), and non-use of either atropine (S4A Fig) or phenylephrine (S4D Fig). No epidural anesthesia at the lumbar level (S5B Fig), laparoscopic surgery (S6A Fig), and lateral positioning during surgery (S6B Fig) were also identified as the risk factor for PONV.

In contrast, patient’s BMI (Fig 2C), duration of anesthesia (Fig 2D), anesthesia start time (morning, noon, evening; Fig 2F), anesthesia method (inhalational or total intravenous anesthesia; Fig 4A), use of sevoflurane (Fig 4C), use of propofol ampules (Fig 4D), use of fentanyl (Fig 4F), use of remifentanil (Fig 4G) use of rocuronium (Fig 4H), use of nicardipine (S4C Fig), use of sugammadex (2 vials; Fig 4I), epidural anesthesia at cervical or thoracic or sacral levels (S5A and S5C Fig), and prone positioning during surgery (S6C Fig) did not have any clear relationship with PONV.

### Results of machine learning modeling

We conducted experiments to show the performance of the model. We used three metrics: precision, recall, and f-score. The definitions are below. *P* and *N* represent numbers of positive (PONV) and negative (no-PONV) samples, respectively. *TP* (true positive) and *TN* (true negative) are the numbers of samples correctly predicted as PONV and no-PONV, respectively. Likewise, *FP* (false positive) and *FN* (false negative) are the number of samples incorrectly predicted as PONV and no-PONV, respectively.


$$precision=\frac{TP}{TP+FP}$$

$$recall=\frac{TP}{TP+FN}$$

$$Fscore=\frac{2TP}{2TP+FP+FN}$$

The results are shown in Table 4. We compared the predictive model (LightGBM) with the k-nearest neighbor algorithm. The precision results of k-NN and our model were very close. In contrast, Our recall was significantly superior to k-NN. While k-NN failed to detect PONV, our model detected PONV more correctly. Therefore, our F-score was better than the k-NN.

**Table 4:** Results of k-NN and LightGBM. The bold represents the best value.

	Precision	Recall	F-score
k-NN (k = 1)	0.2	0.21	0.21
k-NN (k = 3)	0.27	0.12	0.17
Ours	0.26	0.57	0.35

Furthermore, we carried out experiments using cross-validation. We used ten splits. Thus, each set consists of 10% training, 20% validation, and 70% test data. The results are shown in Table 5. The average and standard deviation of the F-score were 0.34 and 0.02. Thus, the performance of the method was stable.

**Table 5:** Cross-validation results of the machine learning model, LightGBM.

Splits	Precision	Recall	F-score
1	0.25	0.54	0.34
2	0.25	0.58	0.35
3	0.25	0.58	0.35
4	0.23	0.51	0.31
5	0.24	0.58	0.34
6	0.21	0.49	0.29
7	0.25	0.59	0.35
8	0.25	0.57	0.34
9	0.25	0.54	0.34
10	0.24	0.55	0.34
Ave.	0.24	0.55	0.34
Std.	0.01	0.04	0.02

The calibration analysis of the model is conducted. The results are shown in S7 Fig. The KNN method failed to predict probabilities of positive samples of PONV. The GBM relatively predicted probabilities of PONV samples. There are gaps between predicted probabilities and perfect fractions of positives, such as blue and black dashed lines in S7 Fig, respectively. We show the values of the fractions to discuss the accuracy of the predictive models. For instance, the number of test samples predicted to [0.0, 0.2) is 1205, including 30 PONVs. Thus, the fraction is 0.03 approximately. Suppose the classification threshold is 0.4, and the GBM classifies 239 (= 115+124) test samples to PONV correctly. Thus, the accuracy score is approximately 70%. In contrast, the KNN misclassified 297 samples, 86% of the total. Therefore, the accuracy of the GBM model is sufficient.

## Discussion

This study is the first to identify the risk factors of PONV using machine learning-based AI. Our study identified total blood loss during anesthesia as the strongest risk factor for PONV, although its SHAP value was independent of its volume.

In the present study, the likelihood of PONV was greater in patients whose total blood loss during surgery was 1–2,500 ml, and intraoperative blood transfusion correlated with suppressing PONV. These findings suggest insufficient circulating blood volume as a risk factor for PONV. Intraoperative hypotension could also contribute to develop PONV [25–27]. Hypotension may lead to cerebral hypoperfusion and brainstem ischemia that may vomiting centers in the medulla. It is also speculated that hypotension leads to gut ischemia and release of emetogenic substances such as serotonin from the intestine [28]. A previous study identified a significant increase in PONV when systolic blood pressure decreased by 35% from the pre-anesthesia baseline value during gynecological surgery [2]. Furthermore, in the present study, PONV was associated with the use of dopamine during surgery and non-use of atropine or phenylephrine was also relevant to PONV. These circulatory agents may prevent PONV by increasing blood pressure and preventing intraoperative hypotension. However, fluid volume has been considered as a risk factor for PONV, although the findings are controversial. Inadequate fluid volume was reported to induce hypotension [25,26] and increased the prevalence of PONV, while excessive fluid volume caused intestinal edema and was also a risk factor for PONV [27]. In the present study, patients who received total fluid infusion volume of fewer than 1,000 ml were identified as a risk factor for PONV [29].

Previous studies have suggested that the duration of surgery more than 60 min is a risk factor for PONV [30]. Similarly, in this study, the duration of surgery between 60 to 400 min was associated with an increased risk of PONV. However, longer duration of surgery (> 400 min) was not correlated to develop of PONV. This may be due to the small number of patients who underwent surgery longer than 400 min, which resulted in poor power of analysis in the AI [31].

Collectively, the relationship between total blood loss and PONV in the present study should take into account changes in circulating blood volume that are influenced by the duration of surgery, infusion volume, and transfusion volume. In this study, we did not investigate the relationship between changes in circulating blood volume and PONV, so further research is needed.

Duration of anesthesia has been considered as a risk factor for PONV, since the duration of anesthesia exposed to emetogenic stimuli like volatile anesthetics may contribute to developing PONV [32]. However, in the present study, machine learning-based AI did not identify the duration of anesthesia as a risk factor for PONV. This finding suggests that the surgical stress itself, rather than the duration of anesthesia, may be a risk for PONV. However, the duration of anesthesia includes not only the time required for anesthesia induction and emergence but also the time required for patient positioning before and during surgery. The time to complete positioning is different for each surgery, and the time without added surgical invasion is different for each surgery. This difference in time for repositioning may bias the results.

It is well recognized that the use of inhalational anesthetics or opioids develop PONV [10,12,33,34], while the use of propofol reduces the incidence of PONV [35]. Similar to the previous findings, the present study identified the use of desflurane for the maintenance of anesthesia and the non-use of propofol prefilled syringe which was used for induction and maintenance of anesthesia as risk factors for PONV. Sevoflurane has been also regarded as a strong risk factor for PONV. Numerous studies have compared the effects of desflurane and sevoflurane on PONV, with conflicting results. Macario et al [36]. showed no difference in PONV frequency between desflurane and sevoflurane use, while some studies reported an increased incidence with desflurane compared to that observed with sevoflurane use [33,34]. The present study revealed that the use of sevoflurane for the maintenance of anesthesia was not a risk factor for PONV. Intraoperative and postoperative use of fentanyl is also regarded as a strong risk factor for PONV [32]. However, the present study revealed that the use of fentanyl for the maintenance of anesthesia was not a risk factor for PONV. Although postoperative use of opioid is considered as risk factor for PONV in adults, and perioperative use of opioid contribute to increasing in the prevalence of PONV in children, it is still under debate whether the intraoperative use of opioid by itself could lead to the developing PONV. Our findings suggest that intraoperative use of fentanyl by itself does not increase the prevalence of PONV at least in adult patients.

Laparoscopic surgery was also identified as the risk factor for PONV, which is consistent with previous reports [37,38]. The etiology of PONV in laparoscopic surgery is not fully understood, but is attributed to residual pneumoperitoneum after CO_2_ insufflation and positional changes [39]. We also identified lateral positioning to be a risk factor for PONV. This finding supports the previous findings that PONV was more common in patients who underwent hip joint surgery [40,41].

### Limitations

Although the present study identified several risk factors for PONV by machine learning-based AI, it is possible that the machine learning-based AI learns spurious relationships, and this possibility cannot be completely ruled out. Thus, further research is needed to prevent spurious relationships. The mechanism by which these risk factors increase the risk of PONV is also not fully understood. In addition, in this study, patients who received antiemetic prophylaxis including droperidol were excluded from the analysis. Patients receiving antiemetic prophylaxis may be patients who are suspected of developing PONV before surgery, and if data are analyzed excluding these patients, the prediction model of the study or the external validity of the risk factor identification may be limited. Furthermore, this study does not evaluate the effects of steroids used during anesthesia, which may reduce the incidence of PONV. Additionally, in this study, the analysis for patients with long surgical times (more than 400 hours) and patients with large blood loss (more than 2500 ml) may be inaccurate. Generally, when the surgery time is long, the patient’s condition may be very poor after surgery. For example, the patient may be in severe pain after surgery, the patient’s level of consciousness may remain low, or the patient may be sedated due to the use of strong analgesics. Therefore, it is possible that the occurrence of PONV was not accurately measured, leading to bias in the results. This may also be due to the small sample size of these patients, but further research is needed. In addition, this study did not examine the interaction of each risk factor. In our study, external validation was not possible because the research design was designed as a single-center study. External validation is necessary to determine whether the predictive model used in the study works well, so further research is needed to determine whether the predictive model we used can be used in other medical facilities. Additionally, the analysis method used in this study does not allow calculation of risk increase rates or risk ratios. This is a limitation of this study.

## Conclusions

Although intraoperative total blood loss was identified as the risk factor most closely associated with PONV, it may correlate with the duration of surgery, total fluid infusion volume, and insufficient circulating blood volume. The present results support previous findings that anesthesiologists should proactively develop PONV prophylaxis, such as the use of antiemetic agents and non-use of desflurane, for patients with universal risk factors for PONV (sex and age). The study also suggested that PONV can also be prevented by shortening the duration of surgery and optimizing fluid management. Further research is required to clarify the mechanisms of how each risk factors develop PONV.

## Supporting information

S1 Fig Patient flow diagram. (PDF)

S2 Fig ROC curves. Performance evaluation of the two machine learning models, measured using a ROC curves. GBM; LightGBM, KNN; *k*-nearest neighbor.
(PDF)

S3 Fig Mean absolute SHAP value of each feature. (PDF)

S4 Fig Relationship between the use of cardiovascular agents and PONV in SHAP value. The X-axis represents the numbers of (A) atropine ampules (0.5 mg per ampule), (B) dopamine prefilled syringes (50 mg per syringe), (C) nicardipine ampules (2 or 25 mg per ampule), and (D) phenylephrine ampules (1 mg per ampule) used during anaesthesia. The Y-axis represents the risk of PONV. SHAP value above 0 is related to the risk of PONV.
(PDF)

S5 Fig Relationship between level of epidural anaesthesia and PONV in SHAP value. The X-axis represents (A) cervical or thoracic (0: no, 1: yes), (B) lumbar (0: no, 1: yes), and (C) sacral (0: no, 1: yes). The Y-axis represents the risk of PONV. SHAP value above 0 is related to the risk of PONV.
(PDF)

S6 Fig Relationship between other factors and PONV in SHAP value. (A) Relationship between laparoscopic surgery and PONV in SHAP value. The X-axis represents 0: non-laparoscopic surgery, 1: laparoscopic surgery. (B and C) Relationship between patient positioning during surgery and PONV in SHAP value. The X-axis represents (B) lateral position (0: no, 1: yes), and (C) prone position (0: no, 1: yes). The Y-axis represents the risk of PONV. SHAP value above 0 is related to the risk of PONV.
(PDF)

S7 Fig Results of calibration analysis. The fractions of positives are defined as the ratios of the numbers of PONVs and samples whose probabilities are [0.0, 0.2), [0.2, 0.4), [0.4, 0.6), [0.6, 0.8), and [0.8,1.0], respectively.
(PDF)
