# Supplementary figures and images for: Machine learning-based identification of the risk factors for postoperative nausea and vomiting in adults
Source: PLoS One. 2024 Aug 15;19(8):e0308755. doi: 10.1371/journal.pone.0308755 (PMC11326632; doi:10.1371/journal.pone.0308755)

**Figure S1.**

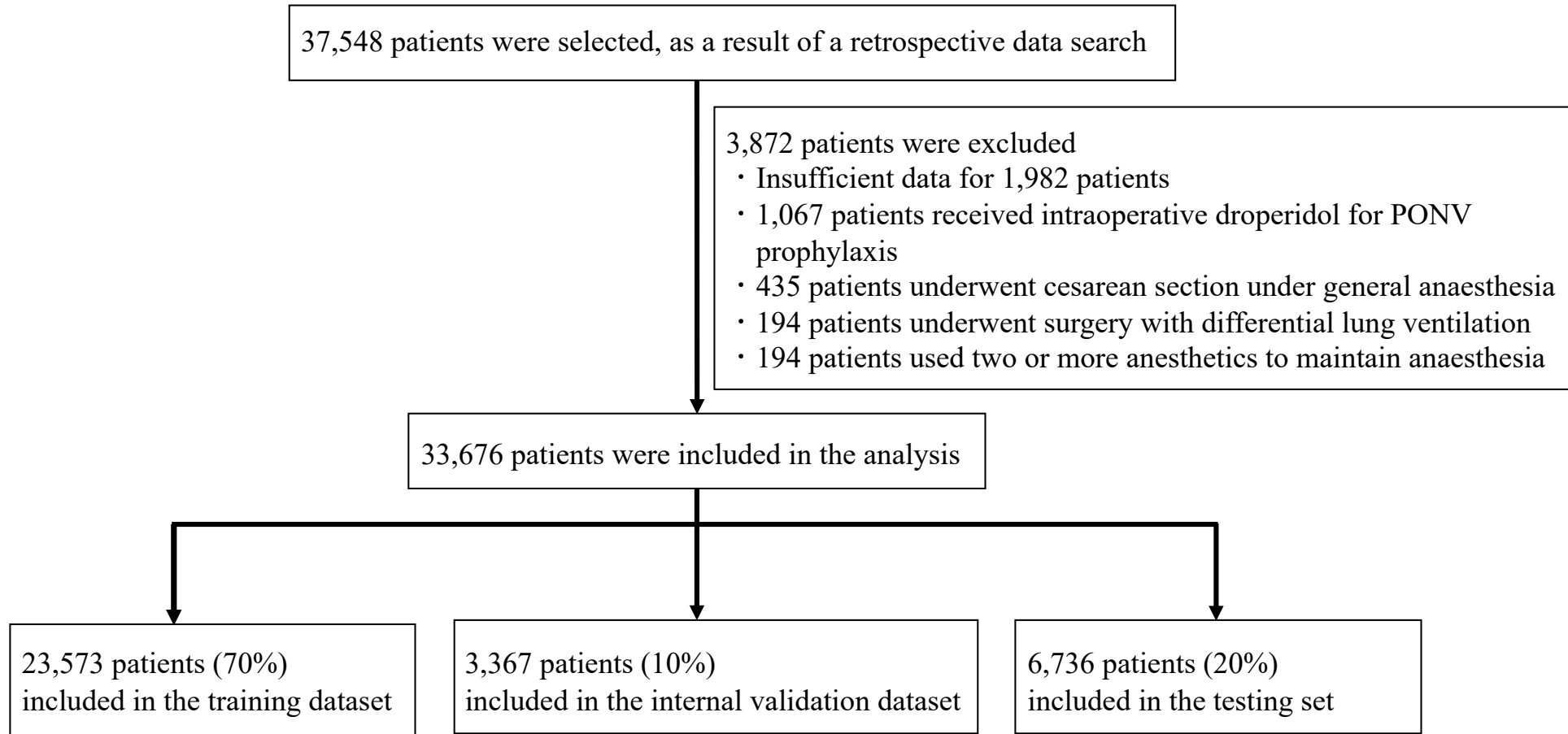

Supplement: S1 Fig — (PDF) [file pone.0308755.s001.pdf]

**Figure S2**

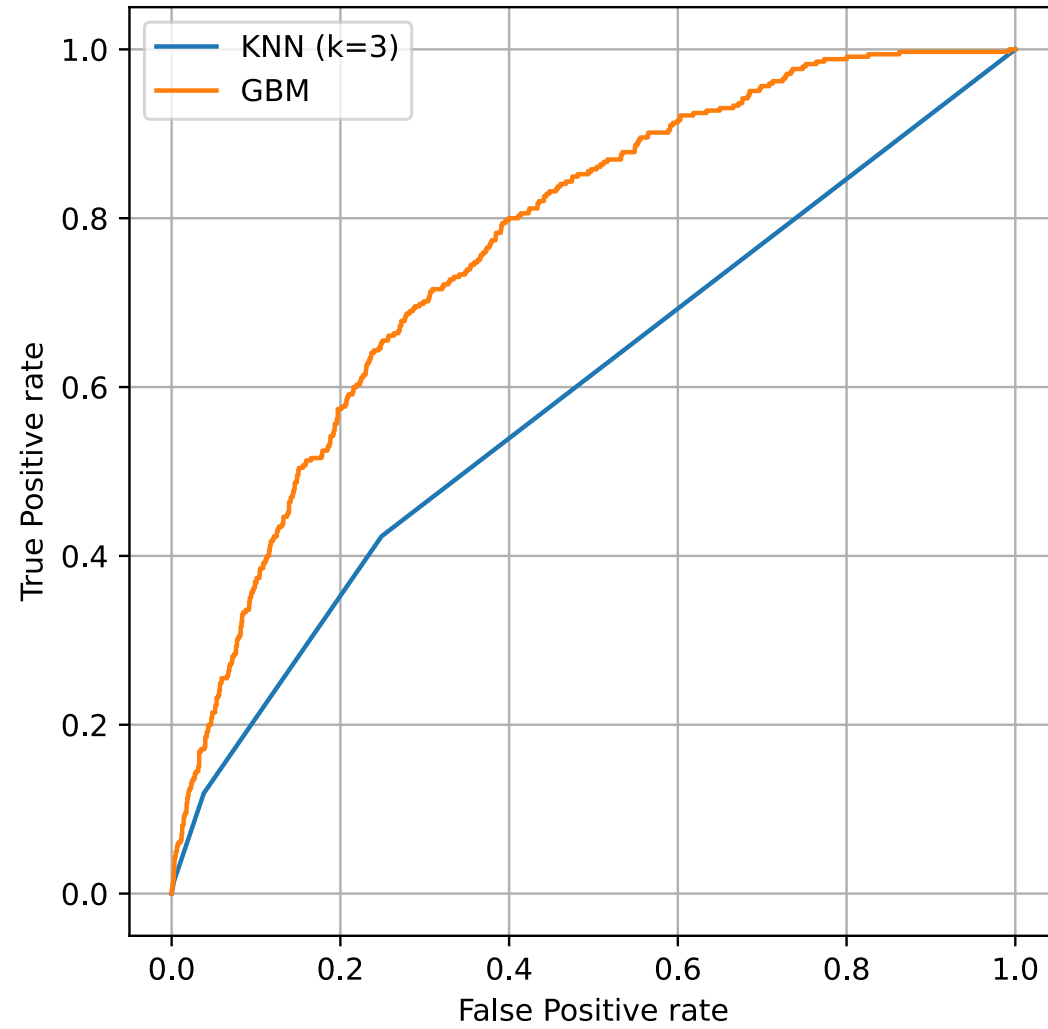

Supplement: S2 Fig — Performance evaluation of the two machine learning models, measured using a ROC curves. GBM; LightGBM, KNN; k-nearest neighbor. (PDF) [file pone.0308755.s002.pdf]

Figure S3

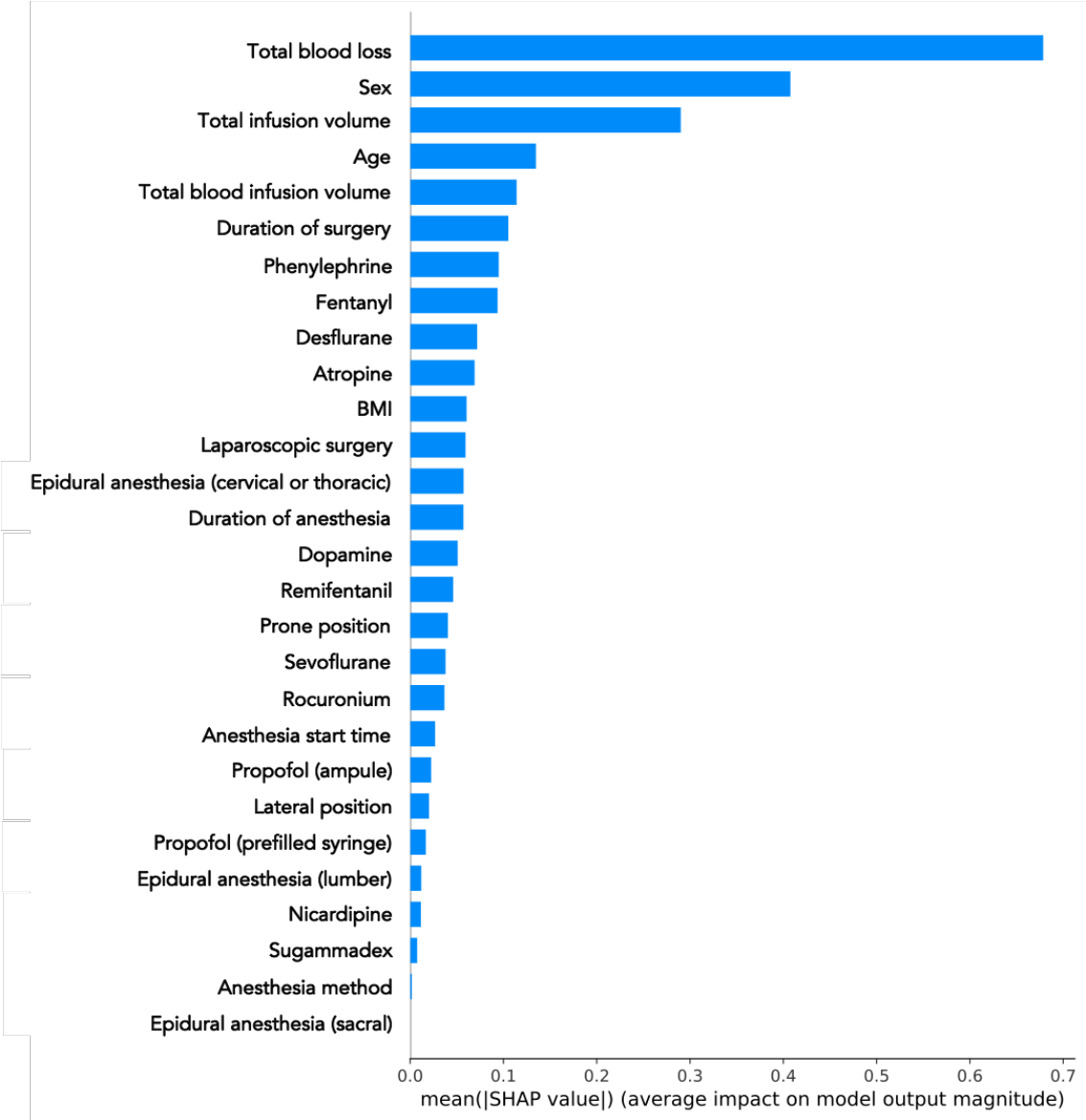

Supplement: S3 Fig — (PDF) [file pone.0308755.s003.pdf]

Figure S4

(A) Atropine

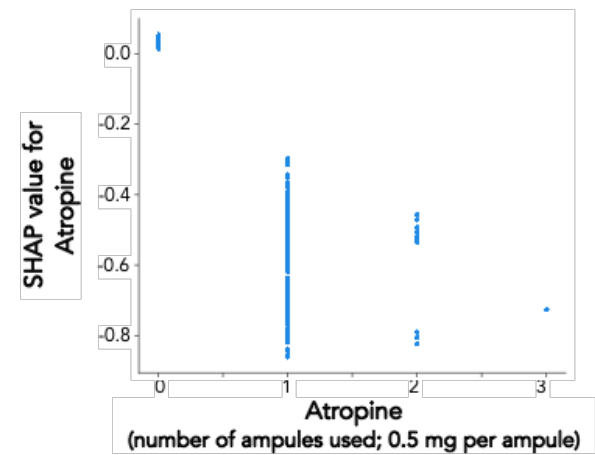

(B) Dopamine

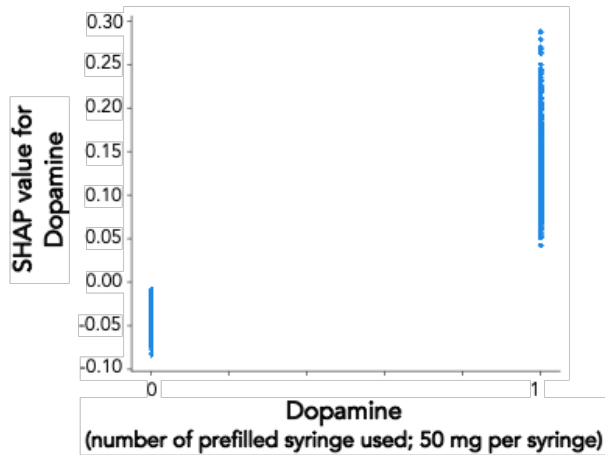

(C) Nicardipine

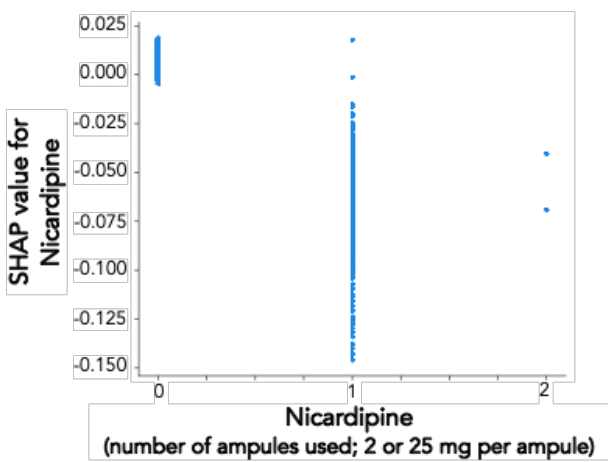

(D) Phenylephrine

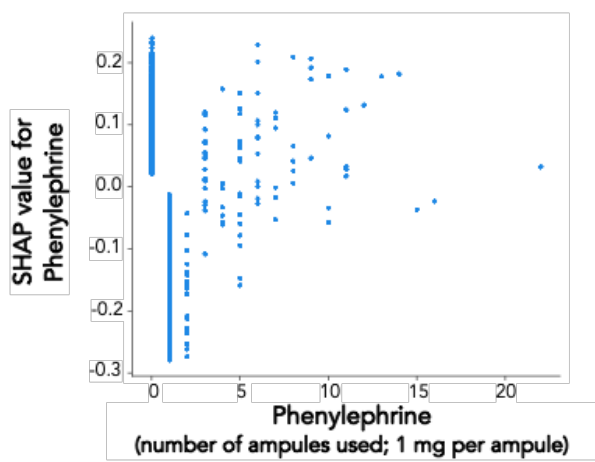

Supplement: S4 Fig — The X-axis represents the numbers of (A) atropine ampules (0.5 mg per ampule), (B) dopamine prefilled syringes (50 mg per syringe), (C) nicardipine ampules (2 or 25 mg per ampule), and (D) phenylephrine ampules (1 mg per ampule) used during anaesthesia. The Y-axis represents the risk of PONV. SHAP value above 0 is related to the risk of PONV. (PDF) [file pone.0308755.s004.pdf]

Figure S5

(A) Cervical or thoracic

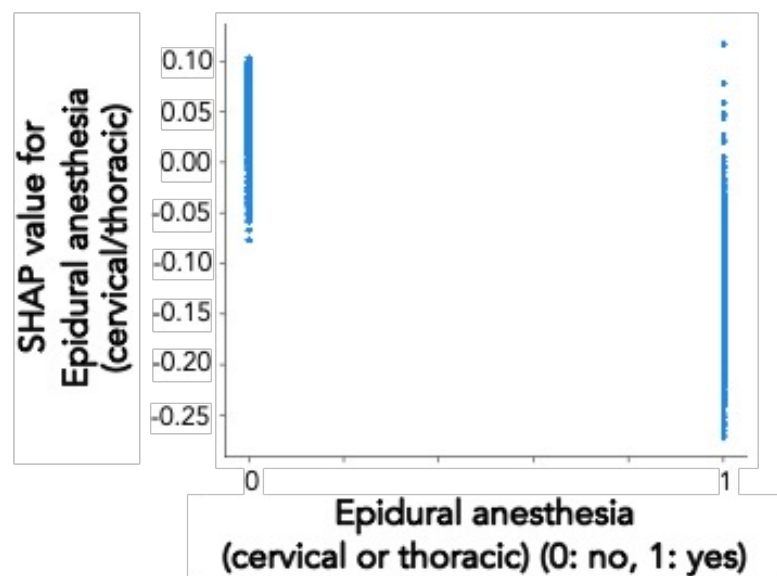

(B) Lumbar

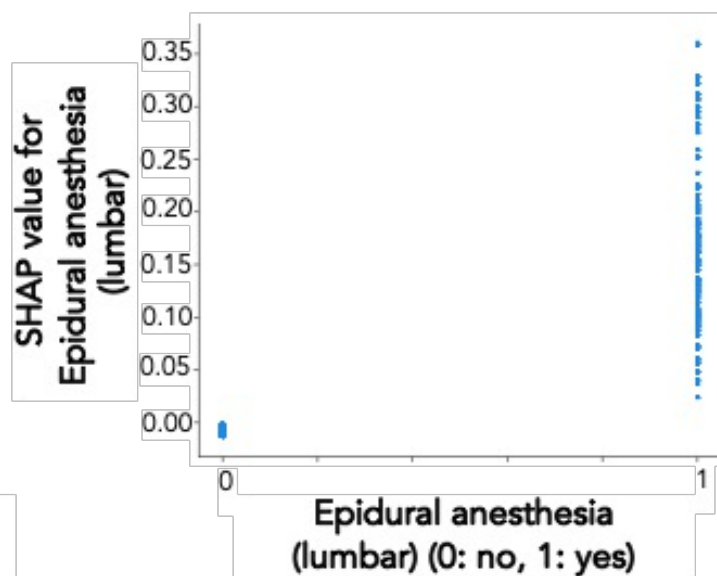

(C) Sacral

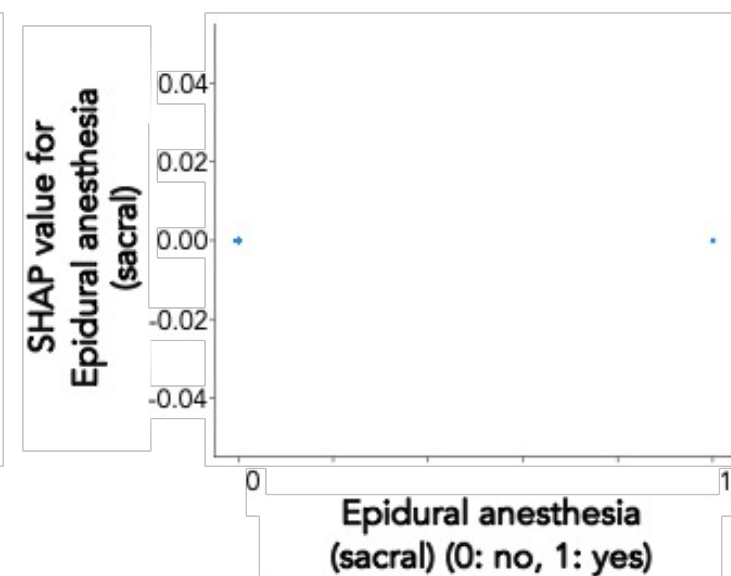

Supplement: S5 Fig — The X-axis represents (A) cervical or thoracic (0: no, 1: yes), (B) lumbar (0: no, 1: yes), and (C) sacral (0: no, 1: yes). The Y-axis represents the risk of PONV. SHAP value above 0 is related to the risk of PONV. (PDF) [file pone.0308755.s005.pdf]

Figure S6

(A) Laparoscopic surgery

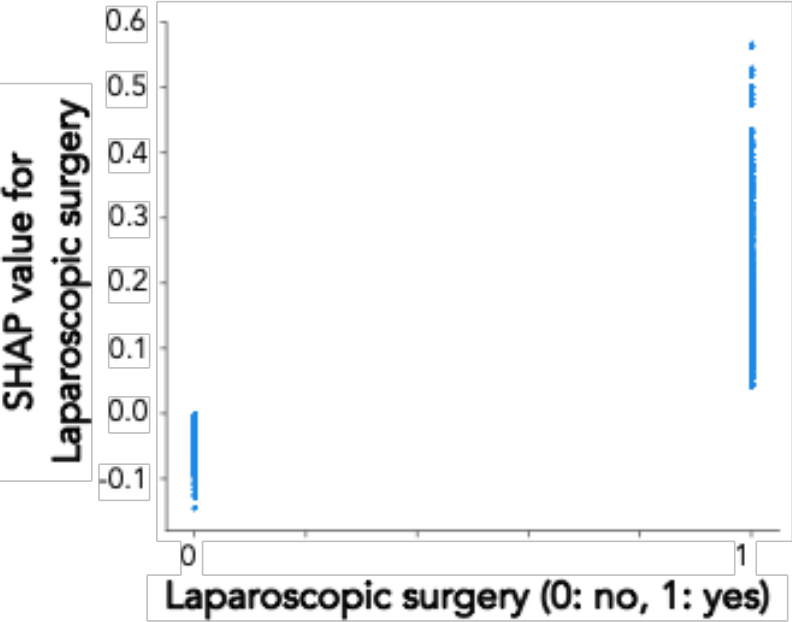

(B) Lateral position

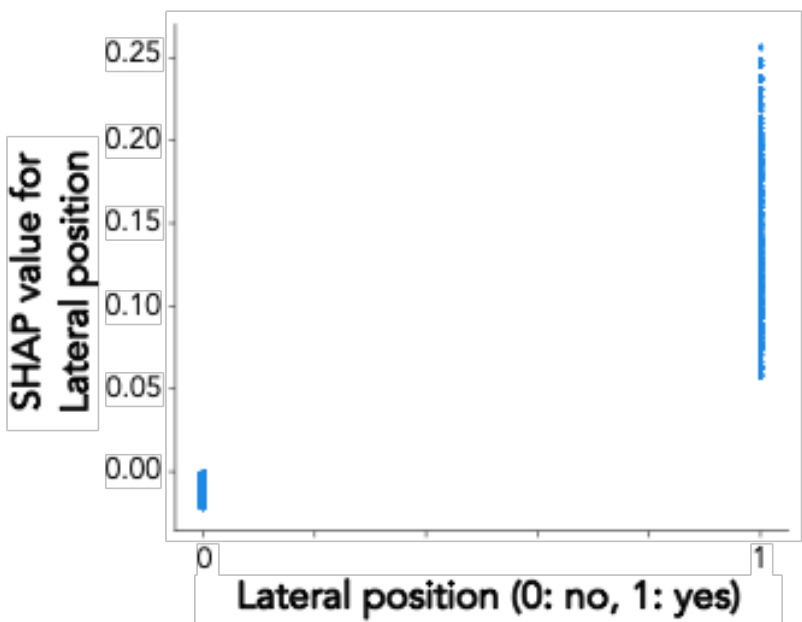

(C) Prone position

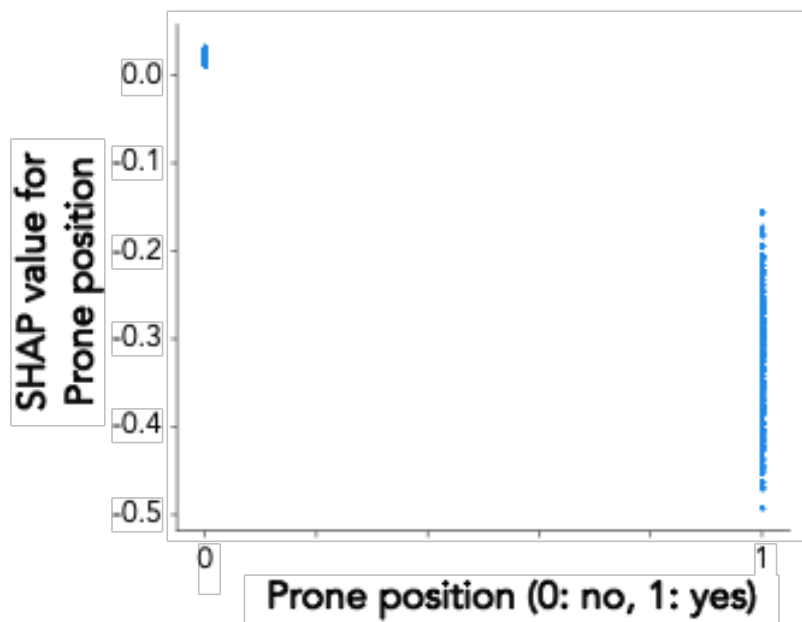

Supplement: S6 Fig — (A) Relationship between laparoscopic surgery and PONV in SHAP value. The X-axis represents 0: non-laparoscopic surgery, 1: laparoscopic surgery. (B and C) Relationship between patient positioning during surgery and PONV in SHAP value. The X-axis represents (B) lateral position (0: no, 1: yes), and (C) prone position (0: no, 1: yes). The Y-axis represents the risk of PONV. SHAP value above 0 is related to the risk of PONV. (PDF) [file pone.0308755.s006.pdf]

Figure S7

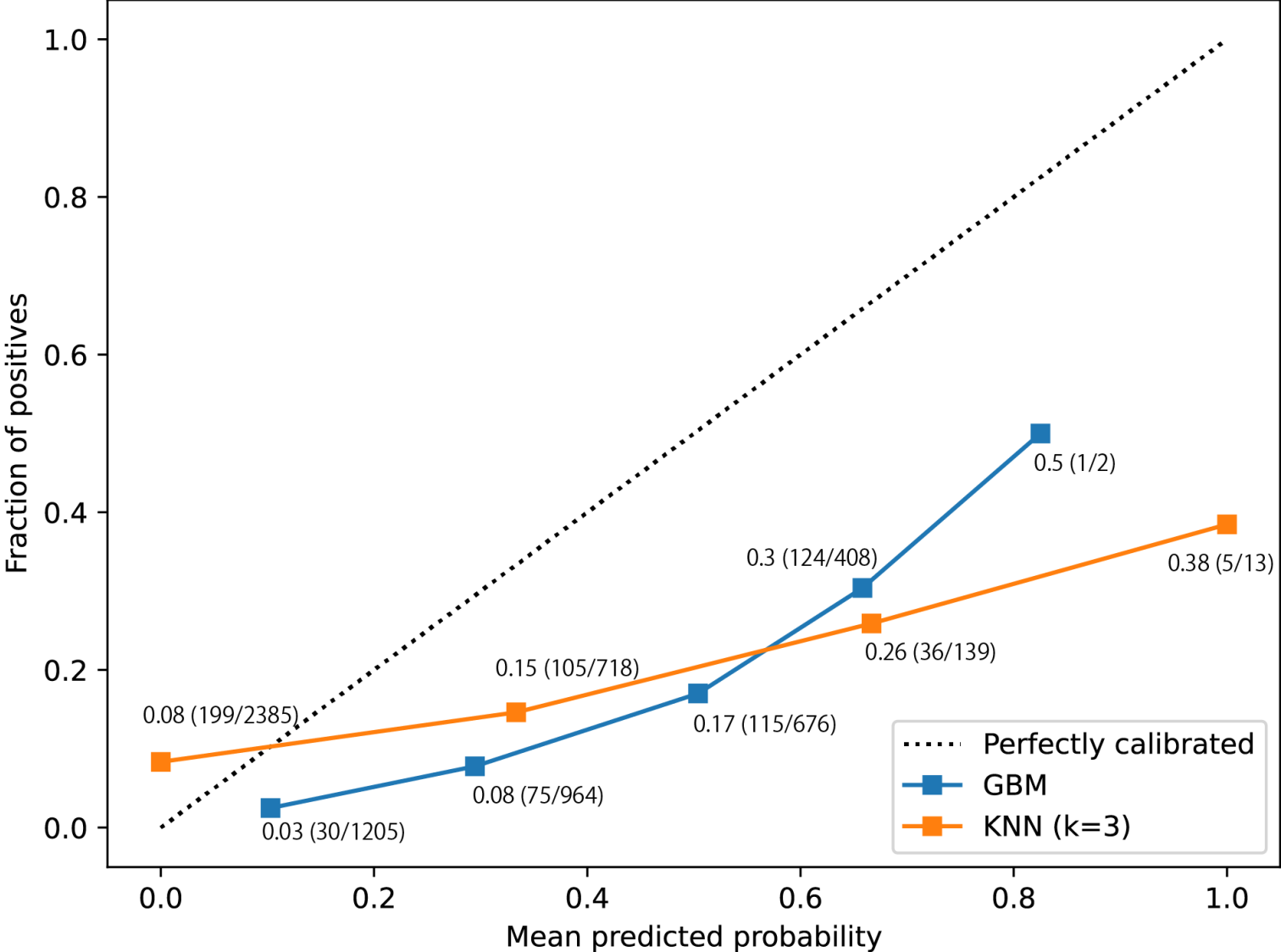

Supplement: S7 Fig — The fractions of positives are defined as the ratios of the numbers of PONVs and samples whose probabilities are [0.0, 0.2), [0.2, 0.4), [0.4, 0.6), [0.6, 0.8), and [0.8,1.0], respectively. (PDF) [file pone.0308755.s007.pdf]
